# The Role of PPAR-γ in Allergic Disease

**DOI:** 10.1007/s11882-021-01022-x

**Published:** 2021-10-25

**Authors:** Julian M. Stark, Jonathan M. Coquet, Christopher A. Tibbitt

**Affiliations:** 1 Department of Microbiology, Tumor and Cell Biology, Karolinska Institutet, Stockholm, Sweden; 2 Centre for Infectious Medicine, Department of Medicine Huddinge, Karolinska Institutet, Stockholm, Sweden

**Keywords:** PPAR-γ, Allergy, Asthma, TH2, ILC2, Lipid metabolism

## Abstract

**Purpose of Review:**

The incidence of allergic diseases such as asthma, rhinitis and atopic dermatitis has risen at an alarming rate over the last century. Thus, there is a clear need to understand the critical factors that drive such pathologic immune responses. Peroxisome proliferator-activated receptor-γ (PPAR-γ) is a nuclear receptor that has emerged as an important regulator of multiple cell types involved in the inflammatory response to allergens; from airway epithelial cells to T Helper (TH) cells.

**Recent Findings:**

Initial studies suggested that agonists of PPAR-γ could be employed to temper allergic inflammation, suppressing pro-inflammatory gene expression programs in epithelial cells. Several lines of work now suggest that PPAR-γ plays an essential in promoting ‘type 2’ immune responses that are typically associated with allergic disease. PPAR-γ has been found to promote the functions of TH2 cells, type 2 innate lymphoid cells, M2 macrophages and dendritic cells, regulating lipid metabolism and directly inducing effector gene expression. Moreover, preclinical models of allergy in gene-targeted mice have increasingly implicated PPAR-γ in driving allergic inflammation.

**Summary:**

Herein, we highlight the contrasting roles of PPAR-γ in allergic inflammation and hypothesize that the availability of environmental ligands for PPAR-γ may be at the heart of the rise in allergic diseases worldwide.

## Introduction

The incidence of allergic disease around the world has increased dramatically in the last decades [1]. There is a pressing need to understand the factors and pathways that shape the development of allergies, in order to develop targeted approaches that may alleviate disease. Allergic disorders such as asthma, rhinitis and atopic dermatitis (AD) are typically associated with strong ‘type 2’ responses marked by cytokines such as interleukin- (IL-) 4 (IL-4), IL-5, IL-9 and IL-13. These induce infiltration of affected tissues by eosinophils and goblet cells, promote the production of immunoglobulin E (IgE) by B cells, which through the activation of mast cells can induce tissue inflammation and cell death [2]. The origins of allergic disease are unclear but genetic and environmental factors both play important roles. Lifestyle factors such as rural or urban living, breast-feeding, exposures to endotoxins, pollutants and pets, and the composition of the microbiota have been shown to affect the chances of developing allergies later in life [3, 4].

Peroxisome proliferator-activated receptor-γ (PPAR-γ) was initially identified in rodents 30 years ago and belongs to the family of nuclear hormone receptors with roles in lipid metabolism, insulin sensitivity, and glucose homeostasis [5]. Its transcriptional activity can be induced by a range of natural and synthetic ligands and is dependent on co-factors such as CCAAT enhancer-binding protein (CEBP) and Retinoid X Receptor alpha (RXRα), which dimerize and bind to high affinity PPAR response elements at target gene promoters and enhancer regions [6–8]. While ligands of PPAR-γ may come from endogenous sources such as prostaglandin secreting myeloid cells or medium-long chain fatty acids in circulation, many foods (fruits, tea and more) and environmental pollutants including phthalates (plastic softeners) are known ligands of PPAR-γ [9, 10]. Initially identified as the master transcriptional regulator of adipogenesis, there is increasing evidence that PPAR-γ modulates responses from lymphocytes including B cells, innate lymphoid cells (ILCs) and CD4^+^ T (TH) helper cells. In this review, we describe the role of PPAR-γ in allergic disease, focussing on its impacts in various cell types.

## PPAR-γ as Negative Regulator of Allergic Inflammation

### Epithelial Cells

Initial studies linking PPAR-γ function to allergic disease focussed on its function in the epithelial compartment, an important site of first-contact with potential allergens such as house dust mite (HDM), pollen and animal dander [11]. Thiazolidinediones (TZDs), a class of PPAR-γ agonists used to treat diabetes, were shown to exert anti-inflammatory affects in models of allergy. The expression of PPAR-γ in airway epithelial cells (AECs) has been repeatedly observed and TZDs have been shown to regulate AEC functions. TZDs were shown to negatively regulate matrix metalloprotease-9 (MMP9) and tumor necrosis factor (TNF) [12•, 13, 14] release from these cells. PPAR-γ was also shown to be upregulated upon exposure of epithelial cells to IL-4, suggesting a form of negative feedback that impaired downstream inflammatory responses. Activation of PPAR-γ downstream of IL-4 impaired release of IL-8 and upregulation of nitric oxide synthase (NOS) [12•]. Murine models of asthma in which TZDs were administered showed that PPAR-γ agonism constrained allergic responses likely by reducing expression of adhesion molecules VCAM-1 and ICAM-1, and chemokines RANTES and eotaxin from AECs [15]. Additionally, the levels of key type 2 cytokines IL-4, IL-5 and IL-13 were all found to be reduced in allergen-exposed mice administered TZDs. More recent studies using mice deficient for PPAR-γ specifically in the AEC compartment demonstrated an exaggerated inflammatory response to the model antigen ovalbumin (OVA). PPAR-γ was found to directly repress transcription of the *MUC5AC* gene [16•, 17, 18], responsible for the secretion of mucus into the airways in allergic inflammation. Release of epithelial-derived alarmins such as IL-25, IL-33 and thymic stromal lymphopoietin (TSLP) was also found to be enhanced in mice where AEC lacked PPAR-γ, as was nuclear factor kappa-light-chain-enhancer of activated B cell (NF-κb) activation [16•]. PPAR-γ may also inhibit vascular smooth muscle proliferation and enhance apoptosis of these cells [19–21]. In addition to the airway epithelium, TZDs also regulate intestinal inflammation by suppressing TNF production and NF-κB activation in the gut epithelium [22–24]. Moreover, patients with inflammatory bowel disease (IBD) have been found to express significantly lower levels of PPAR-γ relative to healthy controls [25–28]. Thus, PPAR-γ activation in epithelial cells has historically been associated with dampening inflammation in response to allergens and other toxins.

### Granulocytes

PPAR-γ agonists have also been shown to reduce neutrophil counts and MPO activity in response to lipopolysaccharide (LPS) challenge possibly via induction of cyclooxygenase-2 (COX2) [29, 30]. Prostaglandin-D_2_ (PGD_2_) and 15-deoxy-Δ12,14-prostaglandin J2 (15d-PGJ_2_), both of which are agonists of PPAR-γ, have been shown to induce neutrophil apoptosis in a dose-dependent manner [31]. The IgE-mediated activation of mast cells, which plays an important role in allergic disease is also suppressed by TZDs [32]. Pioglitazone was able to prevent anaphylaxis induced by IgE and driven by mast cells, when administrated orally. Similarly, exposure of human eosinophils to TZDs leads to increased rates of apoptosis as well as reduced eotaxin-directed migration and binding to ICAM-1 [33, 34]. The functional capacity of eosinophils is also impaired by PPAR-γ activation leading to reduced levels of antibody-dependent cellular cytotoxicity (ADCC), IL-5-induced CD69 expression and eosinophil-derived neurotoxin (EDN) release [35–37]. While treatment with PPAR-γ agonists have largely been shown to negatively regulate the functions of granulocytes, nanomolar-picomolar concentrations of 15d-PGJ_2_ have been shown to enhance eosinophil migration towards eotaxin and activate actin polarisation [38], suggesting that the impacts of PPAR-γ are dependent on ligand availability and the potency of agonist ligands. Taken together, the data suggests that PPAR-γ activation with synthetic or otherwise potent agonists typically negatively regulates granulocyte functions.

### Suppressive Regulatory T Cells

Regulatory T cells are a population of CD4 T cells with immune suppressive functions [39], in which PPAR-γ has been shown to play an important role, in particular in the adipose tissue [40]. The loss of PPAR-γ in the Treg cell population led to a significant loss of these cells from the visceral adipose tissue (VAT) and played a major role in restoring insulin sensitivity [40, 41, 42••, 43–45]. Mechanistically, PPAR-γ appeared to be induced by signals through the TCR in an IRF4-dependent manner, and was together with IRF4, important for expression of the IL-33R on VAT Treg cells [44, 46]. In allergic immune responses, a role for PPAR-γ in Treg cells has also been demonstrated. In a model of allergic rhinitis, administration of PPAR-γ agonists boosted Treg cell function and dampened signs of allergic inflammation [47]. Furthermore, the accumulation of IL-33R^+^ Treg cells in response to HDM allergens was shown to be somewhat dependent on PPAR-γ [48••].

Altogether, a significant body of evidence suggests that PPAR-γ serves as an important regulator of inflammatory allergic responses, by suppressing pro-inflammatory gene expression in epithelial cells, regulating granulocyte activation and by promoting the activity of suppressive Treg cells.

## PPAR-γ as a Driver of Allergic Inflammation

### T Helper 2 Cells

TH2 cells are classically defined by the expression of transcription factors GATA3 and STAT6, and the effector cytokines IL-4, IL-5 and IL-13. Through the secretion of IL-4, IL-5 and IL-13, TH2 cells promote B cell isotype class switching to IgG1 and IgE, eosinophilia and goblet cell metaplasia [49], and thus drive many hallmarks of allergic disease. Genome-wide expression analyses of TH cells in the HDM model demonstrated the upregulation of a number of genes in lung- and airway-infiltrating TH cells [50]. One of these was PPAR-γ, which was also associated with the upregulation of known downstream target genes including *Retnla, Arg1* and *Chi3l3*. Upon further analysis, PPAR-γ was found to be specifically expressed by TH2 cells rather than interferon-γ^+^ (IFN-γ) TH1, IL-17^+^ Th17 or suppressive Treg cells [48••, 51••, 52]. Mice lacking PPAR-γ in the CD4^+^ T cell compartment had reduced eosinophil infiltration and goblet cell metaplasia in response to HDM instillation, both hallmarks of TH2 cell-mediated allergic airway responses [48••, 51••]. Underlying this attenuated response to allergen challenge was a substantial reduction in IL-5^+^IL-13^+^ TH2 cells. Intriguingly, like VAT Treg cells, Th2 cells also express high levels of IL-33R, and this is important for their ability to mediate allergic inflammation [53]. In response to HDM allergens, CD4 T cells lacking PPAR-γ failed to fully upregulate IL-33R and thereby benefit from innate signals produced by lung epithelial cells. In a reduced in vitro system, exposure of TH2 cells to ligands of PPAR-γ drove expression of ST2 (a subunit of the IL-33R), confirming the importance of PPAR-γ in the induction of this key receptor. Evidently, PPAR-γ is also likely to be an important factor for TH2 cell responses in other situations, since mice lacking PPAR-γ in the T cell compartment failed to generate robust TH2 cell responses to nematode infection and were lacking endogenous TH2 cells in adipose tissue [48••]. Evidence that PPAR-γ is highly expressed in human TH2 cells highlights that this nuclear receptor is likely to play a similar role in humans [51••, 54, 55, 56•, 57].

Further insights into the role of PPAR-γ in TH2 cells also comes from CRISPR knockout library screening approaches, which demonstrated a central role for PPAR-γ in regulating both metabolic responses to fatty acids as well as the expression of canonical TH2 cell genes such as *IL5*, *IL13* and *GATA3* [55, 58]. An intriguing aspect of the role of PPAR-γ on TH2 cell responses is that its impact is more evident in tissues rather than in the lymph nodes. The impact of loss of PPAR-γ had equivocal impact on IL-4 expression [48••, 51••], but profound impacts for IL-5 and IL-13 expression, which is preferentially expressed by tissue TH2 cells. Moreover, the impact of PPAR-γ on ST2 expression was evident on lung tissue TH2 cells, but not on TH2 cells in the lung-draining medLNs. It is possible that this reflects the availability of ligands of PPAR-γ in these different niches, with tissues comprising more prostaglandin producing myeloid cell populations, which may potentiate type 2 responses.

### Other TH Cell Subsets

Although studies have pinpointed subset specific expression of PPAR-γ primarily to TH2 cells and some Treg cells, roles for PPAR-γ have also been proposed in other TH cell subsets. Studies have indicated an importance for PPAR-γ in the expression of IL-9 by TH9 cells [56•]. Admittedly, TH9 cells may be considered a highly differentiated fraction of TH2 cells with the ability to produce high levels of IL-5, IL-13 and IL-9 concomitantly. This has been further reinforced by studies showing significant co-expression of *PPARG* and *IL9R* in two independent asthma cohorts [54, 57]. A link between PPAR-γ and signalling through TGF-β receptors [56•, 57], key in the differentiation of TH9 cells [59], also highlights that PPAR-γ may play a particularly important role in promoting IL-9 production by TH cells. In addition, others have shown that follicular TH (TFH) cell responses were affected in a gender-specific manner. This is suggestive of a possible link between estradiol-2 (E2) and PPAR-γ with TZDs having differential effects depending on menstrual cycle stage [60]. If such observations hold true, this may mean that E2 levels regulate PPAR-γ activity in a range of cell types, with some evidence that menstrual cycle phase can influence the course of asthma in women [61]. Taken together, these studies demonstrate that PPAR-γ is expressed by a range of CD4 T cells, including suppressive Treg cells and pro-allergic Th2 cells. Understanding the cell specific effects of PPAR-γ will be critical in establishing effective and safe treatments to combat allergic pathologies. One possibility is that cell-specific transporters of PPAR-γ ligands into cells could help target PPAR-γ ligand administration to some cell types over others and thereby, potentiate the function(s) of those cells specifically. Further work will need to be conducted to identify such mechanisms.

### Type 2 Innate Lymphoid Cells

ILC are analogous to TH cells in the sense that they are capable of expressing a similar range of effector cytokines and controlled by a similar set of transcription factors [62], yet ILC lack a T cell antigen receptor (TCR). ILC2 have been shown to be increased in patients with various allergic disorders including allergic rhinitis, asthma, AD and eosinophilic esophagitis [63–65]. Early studies of ILC using an unbiased single cell approach indicated that ILC2 were enriched for PPAR-γ and this was confirmed in bulk RNA-Seq data of asthmatic patients challenged with allergen [66, 67].

More recent studies have gone on to demonstrate that while PPAR-γ is dispensable for ILC2 development in the bone marrow, its expression was dependent upon exposure to IL-33 in tissues such as the lung [68]. In line with studies of TH2 cells, loss of PPAR-γ reduced expression of ST2 which has been shown to have multiple putative PPAR-γ binding sites in the promoter of *Il1r1* gene [69]. Enforced overexpression of ST2 in PPAR-γ deficient ILC2 almost completely rescued their ability to respond to papain.

A concurrent study indicated that PPAR-γ served to control expression of CD36, thus regulating lipid uptake by ILC2 [68] and again speaking to a role for PPAR-γ in regulating metabolism. ILC2 have been shown to depend on fatty acid metabolism for their function [70], and subsequent studies have clearly elucidated an important role for PPAR-γ in the management and storage of lipids upon activation of ILC2 [68, 71•]. Lipids acquired by ILC2 were stored in lipid droplets, which were regulated by PPAR-γ in conjunction with DGAT1 in response to IL-33 signalling [71•]. Interestingly, switching mice to a ketogenic diet prevented lipid droplet formation in ILC2 and reduced the severity of airway inflammation. PPAR-γ has also been shown to play a role in glycolysis and to be upregulated by the activation of mTORc1 [71•, 72]. Loss of *Arg1*, a downstream target of PPAR-γ, impaired ILC2 proliferation and responses to papain. Moreover, its loss led to profound metabolic impairments particularly with regards to polyamine biosynthesis and glycolysis [73].

Potentially linked to the metabolic programming of ILC and TH cells by PPAR-γ is the expression of programmed cell death protein-1 (PD-1). PD-1 expression is high in both TH2 and ILC2, and PPAR-γ agonists have been shown to increase PD-1 in ILC2 [74]. While PD-1 acts as a clear brake on inflammation [75], studies of the impact of PD-1 on ILC2 function have been equivocal with loss of PD-1 shown to reduce IL-5 and IL-13 in one study, but to have no effect in another [74, 76]. High expression of PD-1 has also been observed on Th2 cells in models of allergen challenge [52] and may be the result of PPAR-γ activation. An intriguing study recently pointed to a role for PD-1/PD-L1 in promoting Th2 cell responses [77]. In that study, PD-L1 expression by ILC2 was shown to promote the differentiation of Th2 cells. Whether agonists of PPAR-γ promote PD-L1 expression on ILC2 has not been analysed and may be one mechanism by which PPAR-γ and PD-1 co-operate to promote type 2 responses.

Taken together, these studies illustrate that PPAR-γ plays an important role in promoting the ILC2 response to alarmins, by regulating the expression of the IL-33R and by exerting striking effects on cellular metabolism.

## A Role for PPAR-γ in Dendritic Cells and Macrophages

The role of PPAR-γ in DC function appears context dependent. An early study depicted that treatment of DC with TZDs led to the induction of anergy in CD4 T cells, with TH1 and TH2 cell differentiation being severely impacted [78]. In a DC-transfer model of allergy, exposure of OVA-pulsed bone marrow-derived DCs (BMDCs) to the synthetic PPAR-γ agonist rosiglitazone reduced T cell proliferation in draining lymph nodes (LNs) and promoted IL-10 release by antigen specific T cells [79]. Other cardinal features of asthma were also found to be reduced including airway eosinophilia and airway hyperreactivity. This was further supported by evidence showing that PPAR-γ-deficient DCs were more activated and potently induced TH cell responses in the lung [80].

Despite PPAR-γ activation in DC having an ability to reduce T cell activation, mice in which PPAR-γ was absent in DC developed impaired type 2 immune responses to HDM and OVA [51••]. PPAR-γ was found to be dispensable for allergen uptake and processing by DC but was important for the migration of CD11b^+^ DC from the lung to draining medLN [51••]. In this, and an older study [81•], IL-4R expression was shown to be important for the upregulation of PPAR-γ in DC, while signals through the IL-33R potentially supported PPAR-γ expression in DC. In support of a role for PPAR-γ in promoting type 2 responses, cDC2 in VAT were specifically shown to express high levels of PPAR-γ where they promoted the accumulation of Treg and insulin and glucose sensitivity [82]. Thus, PPAR-γ activation in DC may inhibit early T cell priming but plays an important role in driving the TH2 cell response thereafter.

In studies of macrophages, PPAR-γ has been identified as a key factor driving the ‘alternative activated’ or ‘M2’ phenotype [83–85]. Signalling through the IL-4R has been shown to rapidly induce PPAR-γ expression in macrophages, which together with STAT6, controls the transcription of hundreds of genes involved in the M2 macrophage response [81•]. Since the M2 phenotype is linked to activation by type 2 cytokines including IL-4 and IL-13, it is not surprising that ILC2 and TH2 cells appear capable of driving M2 development in the lung tissue [86–88]. Observations of increased frequencies of M2 macrophages in the airways of allergic asthmatics compared to healthy controls (> 2.9 fold) also attest to a potentially inflammatory role for PPAR-γ in macrophages and allergic inflammation [89].

However, anti-inflammatory functions for PPAR-γ in macrophages have also been noted. For one, PPAR-γ is important for the development of alveolar macrophages (AM) during fetal development [90]. Loss of PPAR-γ during early hematopoiesis results in a lack of AM, leading to death via severe pulmonary alveolar proteinosis (PAP). Deletion of PPAR-γ later in life also leads to arrested AM development and lethal PAP [90, 91]. Moreover, in bleomycin-induced lung injury models, PPAR-γ promotes the expression of anti-inflammatory cytokines such as IL-10 and hepatocyte growth factor (HGF), which can dampen neutrophil infiltration and fibrosis [92, 93]. Finally, loss of PPAR-γ in macrophages in VAT leads to increased inflammation and insulin resistance in obesity [94]. Thus, PPAR-γ plays a pivotal role in macrophage biology, which by supporting the M2 macrophage phenotype may potentiate certain facets of the type 2 immune response.

Overall, PPAR-γ regulates the functions of hematopoietic and non-hematopoietic cells (see Table 1). Understanding the precise activities of this nuclear receptor in diverse cell types will be important for appropriately targeting its functions in allergic disease.

**Table 1:** Cell-specific effects of PPAR-γ

Cell type	Effect of PPAR-γ activation	References
Epithelial cells	Downregulation of inflammatory response via reduced Nf-κB signaling, TNF and MMP-9 release Repression of MUC5AC Reduced VCAM-1, ICAM-1, RANTES and eotaxin release Suppression of alarmin release	[12•, 14, 16•, 18, 21]
Granulocytes	Reduced MPO and neutrophil infiltration by COX2 activation Induction of neutrophil apoptosis Suppression of mast cell IgE-mediated degranulation	[30–32]
Treg cells	Required for adipose tissue Treg PPAR-γ agonists increase Treg frequency in allergy	[40, 44, 45, 47]
TH2 cells	Important for ST2 expression, differentiation in tissue and production of IL-5 and IL-13 Likely important in metabolic programming	[48••, 51••, 52]
Other TH subsets	TH9 cells require PPAR-γ for differentiation Suggested link between TGF-β signaling driving PPAR-γ in TH9 cells Gender specific regulation of TFH cell responses driven by E2 cooperation with PPAR-γ	[56•, 57, 60]
ILC2	Important for ST2, CD36 and PD-1 expression Controls lipid droplet formation and is required for full function in lung tissue	[68, 69, 71•, 74]
Macrophages	Key in M2 macrophage differentiation and works together with STAT6 in regulating gene transcription Required for development and maintenance of alveolar macrophages	[51••, 81•, 89, 90]
Dendritic Cells	Regulates gene transcription in conjunction with STAT6 Important for migration of CD11b^+^ DC to lymph and the generation of a TH2 cell response	[51••, 81•]

## Future Perspectives of PPAR-γ in Allergic Disease

Much of the early work on PPAR-γ focussed on its ability to limit pro-inflammatory gene expression from epithelial cells and macrophages and a great deal of preclinical research demonstrated that TZDs reduced allergic inflammation in preclinical models. Ultimately, in trials of asthma and COPD, TZDs failed to dampen lung inflammatory responses [95, 96]. Over the past 10 years, it has become clear that PPAR-γ is a critical component of the type 2 immune response to allergens. In particular, PPAR-γ is important in driving allergic responses in tissue, for instance by promoting IL-33R expression on TH2 cells and ILC2, which allows for increased responsiveness to the alarmin, IL-33. We propose that this nuclear receptor could be an important link between the environment and allergic disease. Analogous to the function of the aryl hydrocarbon receptor (AHR) in potentiating TH17 cells and IL-22 production [97, 98], PPAR-γ may be a critical environmental sensor that modulates allergic immune responses. Ligands of PPAR-γ are present in common foods including fruit and tea, but the industrial revolution has also led to an increase in synthetic ligands [99, 100]. One class of synthetic molecules known as phthalates [101, 102], which are added to plastics for increased flexibility, has been implicated in asthma for some time [102] and has been shown to potentiate allergic disease in preclinical models of allergy [103–106]. Phthalates have become ubiquitous in our world and can be readily detected in bodily fluids including blood and urine [107]. Understanding the extent to which environmental PPAR-γ ligands (which can be both agonistic and antagonistic) are responsible for the precipitous rise in allergic disease could lead to targeted therapeutics or the implementation of strategies that mitigate exposures to some of these compounds.
